# Stress of Dialysis Nurses—Analyzing the Buffering Role of Influence at Work and Feedback

**DOI:** 10.3390/ijerph17030802

**Published:** 2020-01-28

**Authors:** Maren Kersten, Sylvie Vincent-Höper, Albert Nienhaus

**Affiliations:** 1 Department of Occupational Medicine, Hazardous Substances and Public Health, Institution for Statutory Accident Insurance and Prevention in the Healthcare and Welfare Services, 22089 Hamburg, Germany; maren.kersten@bgw-online.de (M.K.); albert.nienhaus@bgw-online.de (A.N.); 2 Center of Applied Sciences of Health, University of Lueneburg, 21335 Lueneburg, Germany; 3 Department of Work and Organizational Psychology, Universität Hamburg, 20146 Hamburg, Germany; 4 Competence Centre for Epidemiology and Health Services Research for Healthcare Professionals (CVcare), University Medical Centre Hamburg-Eppendorf (UKE), 20246 Hamburg, Germany

**Keywords:** dialysis nurses, resources, buffering role, feedback, influence at work, Job Demands–Resources model, stress

## Abstract

Dialysis nurses face complex and demanding working conditions. Due to demographic changes, the number of dialysis patients has increased, while the number of skilled personnel is expected to decrease, leading to tremendous increases in quantitative demands in the near future. Against the background of increasing workload, focusing on the provision of job resources is considered a promising approach because resources can buffer the negative effects of job demands. The aim of this study is to investigate whether different job resources—in particular influence at work and feedback—play a buffering role in the relationship between job demands and employee well-being. The study used a cross-sectional paper–pencil survey design. Data were collected from 951 dialysis nurses working in dialysis facilities in Germany between October 2010 and March 2012 using validated measures of quantitative job demands, job-related resources (influence at work and feedback), and cognitive stress symptoms. To test the moderating role of resources, we applied hierarchical regression analyses. The findings indicate that feedback buffers the relationship between quantitative demands and well-being; that is, the positive relationship between quantitative demands and cognitive stress symptoms was weaker when feedback was high. However, we found no buffering role of influence at work. The results suggest that feedback is a promising resource that may buffer the negative impact of quantitative demands on well-being of dialysis nurses. The findings offer new approaches for training nurses and implementing a feedback culture.

## 1. Introduction

Nurses working in dialysis units face complex and demanding working conditions. Work tasks include prolonged and close contact with chronically ill patients [1,2,3], confrontation with the suffering and death of patients [4,5], and the use of complex technology [6,7,8]. Other sources of stress are a reduction in staff [1,9] and few opportunities to participate in decision-making processes [10].

A number of studies revealed that workload is a substantial stressor for employees working in dialysis units [10,11]. Dialysis nurses experience high levels of pressure and stress [5,10,11,12,13]. Moreover, dialysis nurses perceive that they lack time for patient care [7]. A parallel development is that at the same time the number of dialysis patients has increased due to an increased life expectancy and that we observe an increasing shortage of skilled nurses [10,14,15]. These developments decrease the nurse–patient ratio and will further increase the already high levels of demands placed on nurses working in dialysis units [1,9,16]. Against this background, dialysis facilities must seek ways to keep their employees motivated and healthy on a long-term basis.

In addition to potentially stressful work characteristics, studies have also revealed several positive work characteristics prevalent in dialysis settings. For example, dialysis nurses perceive their work as meaningful and are interested in professional knowledge [10]. They experience opportunities to do something for other people and appreciate the freedom to make their own judgments [3]. Moreover, dialysis nurses value the high levels of responsibility that they assume [10].

Positive work characteristics are referred to as job resources. In recent years, a growing body of psychological research has moved away from merely investigating the negative effects of stressors to focusing on the role of job resources. These theories aim at providing insight into the characteristics of the job that enable individuals to maintain their health and well-being despite high demands [17]. The conservation of resources (COR) theory [18,19] highlights the importance of obtaining and protecting resources to address stressful situations and maintain well-being. Resources not only have a direct positive effect on well-being but also attenuate job stressors and the associated negative effects on well-being [20].

Against the background of increasing demands, such as workload, focusing on the provision of resources may be a promising approach because resources are able to buffer the negative effects of job demands [21,22]. There is a large body of research demonstrating that job demands (e.g., time pressure) are related to impaired well-being [23,24]. However, in practice, it is often difficult to redesign structural demands because they are mostly determined by external factors, such as demographic changes. The good news is that resources offer opportunities for designing work in a health-promoting way. Empirical evidence suggests that resources, which can buffer the effects of job demands, have not yet been used to full capacity in dialysis units [10,25].

Research provides evidence for the notion that job control buffers the effect of quantitative demands on well-being [26,27]. Furthermore, the recent literature suggests that feedback may be an important resource contributing to the well-being of nurses [28]. Feedback is relevant for self-evaluation and the achievement of work goals [29], and we therefore assume that it helps to address challenging demands. However, there is a paucity of studies examining the relationship between feedback provision as a resource and employee well-being. In addition, there is evidence that dialysis nurses receive relatively low levels of feedback. Kersten et al. found that dialysis nurses reported lower levels of actually experiencing feedback from colleagues and supervisors compared to geriatric and hospital nurses [25]. Other studies also suggest that there may be a lack of feedback provision in dialysis settings. Di Iorio et al. note that nurses working in dialysis units receive insufficient support on uncertainties, perceive inconsistent expectations, and lack involvement [30]. Ridley et al. reported that 72% of the surveyed nephrology nurses perceived that their chief nursing officers were not accessible and visible to them [31].

So far, there is no systematic framework for dialysis settings that explains the buffering role of different resources on the negative effects of certain stressors. We assume that both structural resources (e.g., job control in terms of influence at work) and social resources (e.g., feedback) can buffer the positive relationship between quantitative demands and employees’ perceived stress. Therefore, the aim of the study is to investigate whether influence at work and feedback as two different types of job resources play a buffering role in the relationship between quantitative demands and impaired employee well-being. By answering this research question, we will advance the understanding of the complex interplay between job resources and job demands in dialysis settings. Analyzing interactions between quantitative demands and different resources increase our insight into the processes leading to the experience of stress. Moreover, as there is a paucity of studies examining the role of feedback as a resource, we aim to close this research gap. Support for the buffering role of job resources is also of practical value because it suggests that increasing dialysis nurses’ levels of job resources may help to attenuate the negative effect of job demands and stress, thus preventing employees from developing high stress levels. Therefore, the findings of the study may have important implications for how to (re)design job resources to counteract the increasing quantitative demands that employees working in dialysis units face.

## 2. Theoretical Background

The Job Demand–Control model (JD–C model) [32,33] is a theoretical framework that highlights the buffering effect of resources on the negative impact of job stressors on well-being. In the JD–C model, control is assumed to be a crucial job resource [34]. According to this model, job control comprises different aspects of discretion, authority, and decision latitude. A core element is employees’ influence on their tasks and task execution as well as on their working conditions.

Studies examining the JD–C model showed that high quantitative demands combined with low control are associated with increased levels of strain and adverse effects on health [27,35,36,37]. However, longitudinal evidence for this interaction effect is limited [38]. Nevertheless, van der Doef and Maes found more evidence indicating that the assumptions of the JD–C model are valid [27].

Johnson and Hall extended the JD–C model by proposing that social support is an additional important resource [39]. The concept of social support has received considerable attention in occupational stress research. Most researchers have used the concept of House [40] to investigate social support [41]. This concept defines social support as emotional, instrumental, and informational aid exchanged through social interactions [42]. Emotional support includes affective participation, empathy, liking, or respect. Appraisal support can be expressed through shared opinions and provides information relevant to self-evaluation, such as a supervisor telling a person that he or she is doing a good job. Informational support includes offering information needed to obtain the job done, and instrumental support includes various sorts of tangible help. The support can be received and assessed from different sources, e.g., supervisors, coworkers, family, and friends [43].

Feedback can be viewed as a specific form of appraisal support that is relevant to self-evaluation. When receiving specific feedback, individuals are able to assess their progress with respect to goal attainment. Moreover, feedback allows the modification of actions or goals and thereby affects the selection of specific strategies or behaviors used to attain these goals [29,44,45].

Haines et al. found evidence that work support buffers the relationship between workload and strain or stress [46]. In their study among (dialysis) nurses, Hayes et al. showed that support from nurse managers and colleagues can act as a buffer against different stressors, e.g., workload, the intense personal relationships with patients, and repeated exposure to patient death [8].

**Figure 1:**
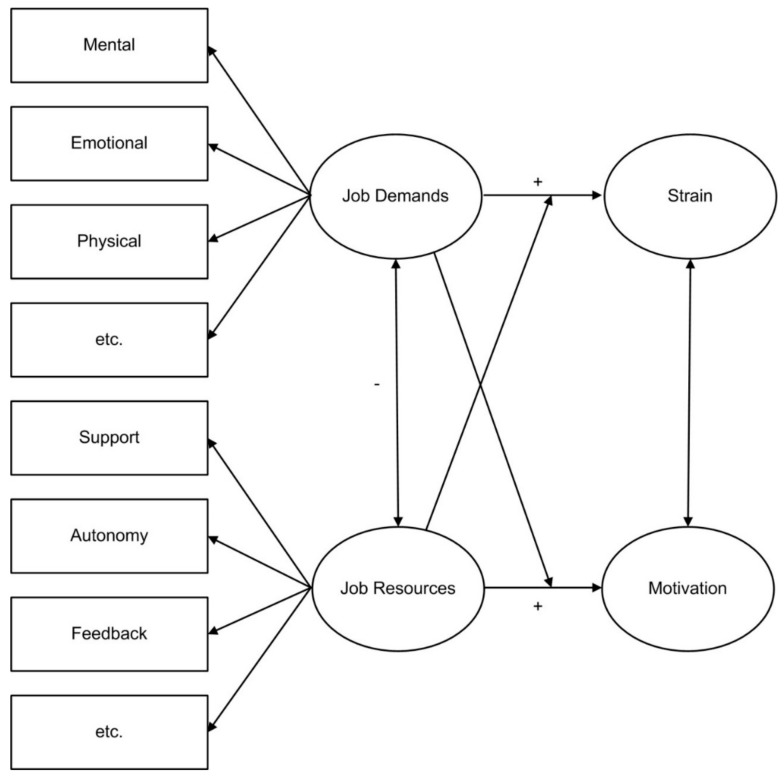
Adapted version of the Job Demands–Resources model.

In current research, the Job Demands–Resources (JD–R) model is a highly popular and widely used theoretical framework for investigating job stress [21]. The JD–R model is an extension of the Demand–Control(–Support) model [33]. The key advantage of this model is that a wide variety of physical, social, cognitive, and organizational factors can be classified in two general categories, i.e., job demands and job resources. According to the JD–R model, a multitude of resources can buffer the relation between job demands und strain, as shown in Figure 1 [44].

Job resources are functional in achieving work goals, in reducing the effect of job demands, and/or in stimulating personal growth, learning, and development [44,47]. According to the JD–R model, receiving feedback is an important job resource. From this perspective, feedback can be interpreted as a means to decrease uncertainty and ambiguity and is assumed to foster learning, thereby increasing job competence.

The JD–R model proposes that high levels of resources are able to buffer the negative effects of high demands, thereby reducing the risk of burnout [21]. Empirical evidence showed that not only resources such as autonomy, social support from colleagues, and a high-quality relationship with a superior but also performance feedback were capable of buffering the impact of work overload on exhaustion. Xanthopoulou et al. found specific interactions between several job demands and job resources (e.g., feedback) that are relevant for home care employees. In this study, social support, opportunities for professional development, and feedback buffered the relationship between workload and exhaustion [22]. The scarce empirical literature provides support for the buffering role of feedback in the relationship between job demands and impaired well-being [21,44,48]. Giesbers and colleagues draw on the buffering role of feedback to develop a theoretical framework for feedback provision in nursing settings [28]. Within this framework, feedback was conceptually linked both to well-being and quality improvement as an additional variable. 

Based on the JD–R model, the present study focuses on job control, operationalized through influence at work and comprising employees’ influence on their tasks and the conditions under which they perform their tasks, as well as feedback, as two different resources that are assumed to be relevant work characteristics in dialysis settings. This study examines the role of these resources in the relationship between quantitative demands and stress. Based on the JD–R model as a theoretical framework and empirical findings, we hypothesize the following:

**Hypothesis** **H1:** *Influence at work buffers the positive relationship between quantitative demands and cognitive stress symptoms.*

**Hypothesis** **H2:** *Feedback buffers the positive relationship between of quantitative demands and cognitive stress symptoms.*

## 3. Materials and Methods

To investigate whether influence at work and feedback play a buffering role in the relationship between job demands and employee well-being, we performed an empirical study in dialysis facilities with a cross-sectional design.

### 3.1. Participants

The study took place in Germany. Three different areas constitute the health system in Germany: outpatient care, hospital sector and outpatient, as well as inpatient rehabilitation facilities. In general, dialysis facilities belong to the outpatient sector, except for dialysis units in hospitals. The dialysis facilities in our study were satellite units, with only one exception. The number of staff differed from facility to facility (13 to 55 persons), as does the number of patients. All facilities cared for patients with all levels of illness.

In total, 1989 dialysis employees in Germany were asked to participate in the study. A total of 1138 employees filled in the questionnaire. Data from 65 participants were excluded from the analyses due to missing values. Therefore, the final sample comprised 1073 participants working in 69 different dialysis facilities.

The study population consisted of two samples that were approached differently. In 20 dialysis facilities, data were collected between October 2010 and March 2011. The majority of these facilities (*n* = 14) were randomly selected. The remaining six facilities belonged to one umbrella organization and were part of a pretest. The pretest was conducted to find out whether the items and scales of the Copenhagen Psychosocial Questionnaire (COPSOQ) instrument were appropriate for employees working in renal units. A total of 191 questionnaires were distributed and 112 dialysis employees participated in the study. The result of the pretest showed that the instrument is adequate for employees working in dialysis settings. Therefore, the participants of the pretest were integrated into the main analyses of this study. In total, 367 employees participated in the first sample.

The second sample was a convenience sample of dialysis facilities of a second umbrella organization of which some facilities participated in the study. Subsequently, the whole organization showed interest in participating. A total of 706 employees working in 49 out of 50 dialysis facilities of the umbrella organization participated in March 2012.

We surveyed not only nurses but also administrative personnel, technicians, social workers, and kitchen personnel. However, in the analyses, we focused on dialysis nurses that were directly involved in care for dialysis patients (*n* = 951). The nurses had different levels of qualifications (certified nurses, trained nurses, other nurses, and nurses in training). Certified nurses received three years of health and nurse training and a special training for dialysis staff of three years. Trained nurses received a one-year training in dialysis work in addition to the three years of health and nurse training, while “other” nurses received no special dialysis training. Nurses in training are apprenticing.

### 3.2. Data Collection

Data were collected using a paper–pencil questionnaire. All items were taken from the COPSOQ. The questionnaire was accompanied by a brief introductory letter, in which the confidentiality and anonymity of the answers were emphasized. Twenty different dialysis facilities were surveyed between October 2010 and March 2011, including six facilities that functioned as a pilot study. A change in the questionnaire after the pilot study was not necessary. Forty-nine other facilities participated in March 2012. Both samples completed the same questionnaire. The response rate across all units was 47.8%.

### 3.3. Measures

In the current study, we assessed quantitative demands as a central demand, as well as influence at work and feedback as job resources. As an outcome variable, we assessed cognitive stress symptoms. All variables were measured using the COPSOQ [49] in its German version [50].

Quantitative demands were assessed with four items. A sample item is “How often do you not have time to complete all your work tasks?”

Influence at work was measured with four items. A sample item is “Can you influence the amount of work assigned to you?”

Feedback was measured with two items asking for feedback from colleagues: “How often do you talk with your colleagues about how well you carry out your work?”; and the supervisor: “How often do you talk with your superior about how well you carry out your work?”

Cognitive stress symptoms were assessed with four items. A sample item is “In the past four weeks, how often did you have difficulties in concentrating?”

Items were scored on a five-point Likert scale ranging from 1 (“never/hardly ever”) to 5 (“always”).

### 3.4. Ethical Considerations

The study was approved by the Hamburg Ethics Committee of the Medical Association (reference number: PV3678). At the start of the survey, detailed study information was provided and participants were informed that completion of the survey implied consent. Participation was voluntary. To ensure anonymity, no names or other identifiers were used.

### 3.5. Data Analysis

We tested the hypotheses by applying correlation and hierarchical regression analyses using SPSS (version 22). To assess the reliability of the used scales, we followed recommendations on Cronbach’s alpha levels outlined by Everitt and Skrondal [51].

To test the moderator hypotheses, we performed multiple hierarchical regression analyses. We controlled for gender (as a dichotomous variable) and age of the employees (as a continuous variable), as well as for part-time vs. full-time (as a dichotomous variable). We z-transformed all variables and assessed quantitative demands as an independent variable, influence at work and feedback as (potential) moderator variables, and cognitive stress symptoms as a dependent variable. To examine the interaction, we created product terms of the predictor and the moderator variables. We analyzed the variation inflation factor (VIF) to test for multicollinearity as a precondition for performing multiple regression analyses. The VIF-values indicated that multicollinearity is not an issue.

### 3.6. Validity and Reliability

The COPSOQ is a well validated instrument for the assessment of psychosocial factors at the workplace and employee strain. The questionnaire was developed at the National Institute of Occupational Health in Denmark. The instrument is broadly based and covers multiple job stress theories (e.g., the Job Demand–Control model, Effort-Reward Imbalance model, Job Characteristics model) [49]. The present study used the validated German translation of the questionnaire [50]. Regarding construct validity, regression analyses showed the theoretically assumed associations [50].

Cronbach’s alpha values of the scales used in this study indicate acceptable internal reliability levels [51]. The scale feedback only contains two items; the intercorrelation of these items is moderate, r = 0.423, *p* < 0.001.

## 4. Results

Descriptive statistics for the study variables are shown in Table 1. Most of the participants were female (87.4%). More than half of the employees (52.9%) were between 30 and 49 years old, 10.3% were younger than 30 years, and 36.8% were older than 50 years. Additionally, 82.3% of the participants were certified nurses, 10% were other nurses, 6.1% were trained nurses, and 1.6% were nurses in training.

Approximately 48.2% of the employees had worked longer than 15 years in their profession, 18.4% had worked between 11 and 15 years, 15.9% between 6 and 10 years, and 17.5% less than 5 years in their profession. Lastly, 45.7% of the participants worked full-time, and 54.3% worked part-time.

The intercorrelations and Cronbach’s alpha coefficients of all variables are shown in Table 2. Quantitative demands were positively related to cognitive stress symptoms (r = 0.224, *p* < 0.001), whereas the job resources’ influence at work and feedback were slightly negatively related to cognitive stress symptoms (r = −0.142, *p* < 0.001; r = −0.072, *p* < 0.05).

**Figure 2:**
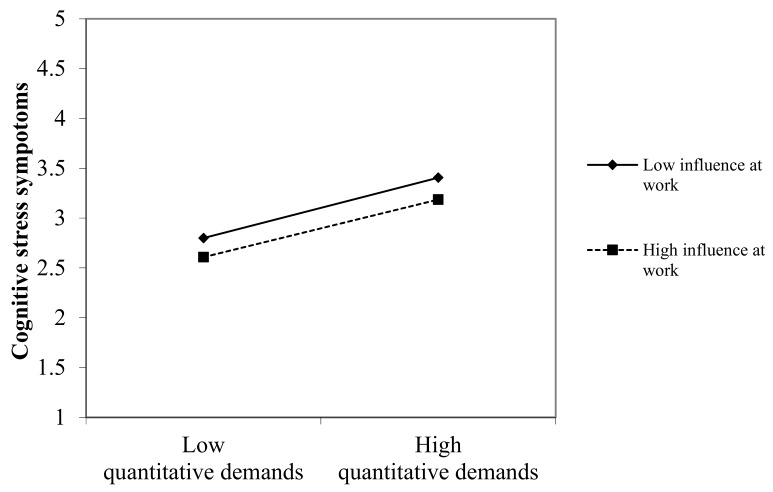
Two-way interaction for the moderator variable: Influence at work.

**Figure 3:**
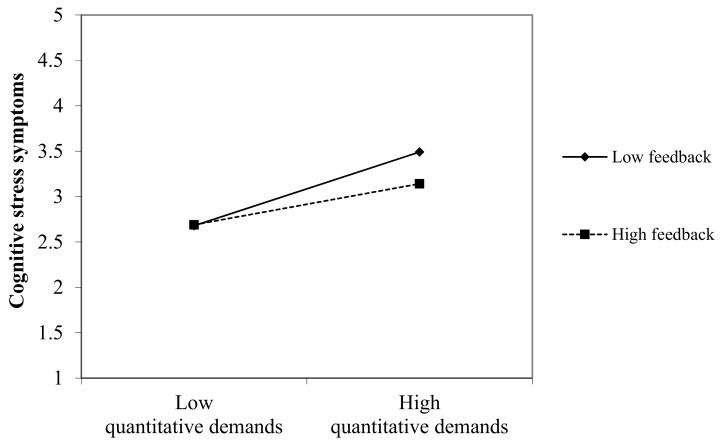
Two-way interaction for the moderator variable: Feedback.

The moderator analysis for the moderator influence at work is shown in Table 3. After controlling for gender, age, and part-time versus full-time, we entered quantitative demands and subsequently the variable influence at work. Lastly, we entered the product term quantitative demands x influence at work. As the criteria variable, we integrated cognitive stress symptoms. The moderator term with influence at work could not explain an additional amount of variance. Thus, we did not find support for Hypothesis 1 that states that influence at work moderates the relationship between job demands and cognitive stress (Figure 2). We then performed the moderator analysis with the moderator feedback in the same way shown in Table 4. Feedback was found to be a significant moderator and the interaction term quantitative demands x feedback explained an additional amount of variance of 1%. Thus, Hypothesis 2 can be confirmed (Figure 3).

## 5. Discussion

The central aim of this study was to analyze whether different job-related resources can buffer the positive relationship between quantitative demands and cognitive stress symptoms of dialysis nurses. More specifically, this study investigated the potential buffering role of influence at work and feedback in the relationship between quantitative demands and stress in dialysis settings. Influence at work was not found to buffer the relationship between quantitative demands and cognitive stress. However, feedback buffered the positive relationship between quantitative demands and cognitive stress symptoms.

The finding that influence at work did not act as a buffer is rather surprising because the interaction between job demands and influence at work constitutes one of the central tenets of the Job Demand–Control model [27,38]. However, de Lange et al. showed in their review of longitudinal studies on the Job Demand–Control–(Support) model that there is only limited evidence for the assumption that high demands combined with low control result in impaired health [38]. Several studies also could not support this interaction effect [52,53]. Bazerman postulated that control only buffers the impact of job demands when employees are actually able to use and to exert control [54]. The levels of influence that employees in dialysis units experience may not allow them to efficiently address their workload. Kersten et al. provided evidence for this notion by showing that employees working in dialysis units report lower levels of influence at work compared to employees in inpatient care for the elderly and sick [25]. In their review of the Job Demand–Control–(Support) model, van der Doef and Maes state that some employees may benefit more from high levels of control compared to others. Our sample comprised nurses with different levels of qualifications. It is conceivable that the interaction between workload and influence at work is only valid for employees with a higher level of qualification [27]. 

In contrast to control, feedback showed a significant buffer effect. The positive relationship between quantitative demands and cognitive stress symptoms is weaker when feedback is high. There is evidence to suggest that feedback is of special relevance for employees’ well-being. This is in line with the theoretical assumptions of Giesbers et al. who developed a theoretical framework for feedback provision in nursing settings [28]. Feedback is crucial for self-evaluation and also for the attainment of work goals, enabling employees to adapt their behavior and develop specific strategies that foster goal attainment [29,45]. In this regard, we assume that feedback is an important resource that helps employees to successfully address job demands.

Although feedback is a generic term that is frequently used, it lacks a theoretical foundation [55]. Future research should provide a clear conceptualization of feedback as a resource in the context of work. A meta-analysis by Kluger and DeNisi indicated that feedback not only yields positive effects, as well as noting that the effect is dependent on the characteristics of the feedback itself, the characteristics of the person giving and the person receiving feedback, and the feedback context [56]. Therefore, we propose that future research should focus on those aspects that influence the effect of feedback. These influencing factors may concern the source of feedback (e.g., supervisor, colleagues, patients, and their relatives), channels of feedback (formal or informal), the type of feedback (directive or facilitative), individuals’ receptivity to feedback (e.g., comfort with feedback, tendency to seek feedback, and behavior change as consequence of feedback), and the organizations’ support for feedback (e.g., feedback culture and coaching to help interpret and use feedback) [57,58].

Another study highlights the processual character of feedback and explains the necessity of evaluating feedback as part of a longitudinal process [55]. Diary studies with a multilevel approach may shed light on the short-, mid-, and long-term effects of feedback processes.

Reducing job demands to keep employees healthy should be a crucial concern for health promotion in organizations. However, in practice, it is often difficult to redesign job demands. The findings of the present study suggest that providing resources, especially feedback, may be an important complementary approach that organizations should consider to attenuate the negative impact of job demands on employee well-being. However, as empirical evidence is scarce, there is a need for further studies that examine the effects of interventions that foster the provision of specific job resources [59]. Empirical findings suggest that personal resources play an important role in determining differential effects of working conditions on well-being. Schaubroeck and Merrit demonstrated that control attenuates the effects of high demands on stress among employees high in self-efficacy, but has contrary effects for those with low levels of job-related self-efficacy [60]. Thus, another concern for further studies should be to incorporate employees’ personal characteristics.

The results not only offer multiple implications for research but also for practice. Nakahara et al. showed that dialysis nurses wish to be more actively involved in treatment decisions and to have more responsibility in treatment care [10]. As it is already common practice in inpatient nursing care, it may be promising to identify work tasks that dialysis nurses can perform more autonomously, e.g., the introduction of patients during ward rounds. Among the professional personnel working in dialysis facilities, nurses are those who spend most of the time caring for patients [8]. Assigning dialysis nurses more responsibility and autonomy contributes to the enrichment of work and enhances nurses’ status (in comparison with doctors).

An improved feedback culture may both attenuate stress symptoms and increase motivation in employees working in dialysis units. Both factors are assumed to be crucial for well-being and retention time. Moreover, this study indicates that enhancing resources, e.g., by implementing an elaborated feedback culture, may be a promising approach to design work characteristics in dialysis settings in a health-promoting way. For dialysis settings, feedback could be fostered through regular (and structured/systematic) appraisal interviews, mentoring, and collegial advice to establish a constructive feedback culture.

### Limitations

Despite several positive aspects, such as the large sample size, several weaknesses of this study should be mentioned. As work characteristics and indicators of well-being were assessed using merely employees’ self-reports, it is possible that common-method variance has inflated the relations between the variables [61]. Future research should take this limitation into account by using multiple sources of information (e.g., supervisors), different assessment methods (e.g., surveys, interviews, and observations), and multiple indicators of employee health and well-being (e.g., self-reports, behavioral, and physiological indices) [20,62].

Moreover, the use of cross-sectional data limits causal inferences regarding the relationships tested. Therefore, future research should examine the buffering effect of job resources in a longitudinal design, in which all variables—preferably theoretically chosen—are measured at various points in time [63,64].

Furthermore, the results of the present study concern the specific group of dialysis nurses in Germany, which show lower levels of feedback compared to geriatric and hospital nurses [25], further restricting the generalization to the working population in other healthcare settings and also to dialysis nurses in other countries. Further studies on the buffering role of job resources in different occupational settings and cultural contexts are needed to strengthen our findings.

The COPSOQ assesses feedback with only two items asking for feedback from colleagues and feedback from supervisors. However, the analyses were based on an overall score for feedback computed by combining feedback from colleagues and feedback from supervisors. This might be the reason why the intercorrelation of the two items is relatively low. For future research, we need a more specific operationalization of feedback considering the different sources (e.g., colleagues, supervisors, patients, and their relatives). Additionally, the assessment of a feedback culture would expand our knowledge in this area. Moreover, an analysis of the underlying mechanisms needed to establish a feedback culture, for example supervisor feedback as a mediator variable, would be an interesting approach. According to Berthelsen et al., it would be important to test the content validity of the feedback construct when establishing a group-level construct based on the individual (or dyadic) level for measurement of cultural phenomenon (e.g., feedback culture) [65].

## 6. Conclusions

The health promotion of employees in specialty areas, such as dialysis, is imperative in the context of increasing workload and the growing global shortage of nurses. The present study investigated the moderating role of job resources in counteracting the demands faced by dialysis nurses. The findings indicate that feedback acts as a buffer against the negative effect of quantitative demands on stress. We suggest that future research would benefit from linking feedback to nurses’ well-being. Furthermore, we argue that dialysis-specific programs aiming at implementing a feedback culture represent a promising approach to reduce stress and promote psychological well-being in the workplace.

## Figures and Tables

**Table 1:** Description of the sample (*n* = 951).

Variable	Categories	*n* (%)
**Gender**	Female	831 (87.4%)
	Male	120 (12.6%)
**Age**	<30	98 (10.3%)
	30–39	134 (14.1%)
	40–49	369 (38.8%)
	≥50	350 (36.8%)
**Type of employment**	Full-time	435 (45.7%)
	Part-time	516 (54.3%)
**On-call duties (per month)**	None	651 (68.4%)
	1–5 times	264 (27.8%)
	>5 times	36 (3.8%)
**Night shifts (per month)**	None	641 (67.5%)
	1–5 times	262 (27.5%)
	>5 times	48 (5.0%)
**Split shifts (per month)**	None	891 (93.6%)
	1–5 times	49 (5.2%)
	>5 times	11 (1.2%)
**Variable shifts (per month)**	None	123 (12.9%)
	1–5 times	269 (28.3%)
	>5 times	559 (58.8%)
**Job experience (years)** **(in dialysis setting)**	≤5	166 (17.5%)
	6–10	151 (15.9%)
	11–15	175 (18.4%)
	16–20	190 (20.0%)
	>20	269 (28.2%)
**Professional group**	Certified nurse	783 (82.3%)
	Trained nurse	58 (6.1%)
	Other nurse	95 (10.0%)
	Nurse in training	15 (1.6%)

**Table 2:** Descriptive statistics, Cronbach’s alphas, and intercorrelations for all study variables.

Scale	*M*	*SD*	1.	2.	3.	4.
**1. Quantitative demands**	2.742	0.596	0.685	−0.138 ***	−0.026	0.224 ***
**2. Influence at work**	3.838	0.793		0.767	0.279 ***	−0.142 ***
**3. Feedback**	3.577	0.804			-	−0.072 *
**4. Cognitive stress symptoms**	3.678	0.774				0.876

*n* = 951; Pearson’s r; * *p* ≤ 0.05; ** *p* ≤ 0.01; *** *p* ≤ 0.001; Cronbach’s alphas appear on the diagonal.

**Table 3:** Hierarchical regression analyses for the moderator variable: Influence at work.

	Cognitive Stress Symptoms	
**Variables**	*Β*	*∆R^2^*
**Step 1** Control variables:		
Gender (employee)	−0.005	
Age (employee)	0.090 **	
Part-time vs. full-time	0.035	
Adj. R^2^: control variables	0.007	
**Step 2**		0.055
QD	0.239 ***	
Adj. R^2^: control variables + QD	0.062	
**Step 3**		0.014
IW	−0.124 ***	
Adj. R^2^: control variables + QD + IW	0.076	
**Step 4**		**0.000**
QD × IW	−0.013	
Adj. R^2^: control variables + QD + IW + (QD × IW)	0.076	

Note: *n* = 951; ** *p* ≤ 0.01; *** *p* ≤ 0.001. QD: quantitative demands; IW: influence at work.

**Table 4:** Hierarchical regression analyses for the moderator variable: Feedback.

	Cognitive Stress Symptoms	
**Variables**	*β*	*∆R^2^*
**Step 1** Control variables:		
Gender (employee)	−0.005	
Age (employee)	0.090 **	
Part-time vs. full-time	0.035	
Adj. R^2^: control variables	0.007	
**Step 2**		0.055
QD	0.239 ***	
Adj. R^2^: control variables + QD	0.062	
**Step 3**		0.007
FB	−0.090 **	
Adj. R^2^: control variables + QD + FB	0.069	
**Step 4**		**0.008**
QD × FB	−0.096 **	
Adj. R^2^: control variables + QD + FB + (QD × FB)	0.077	

Note: *n* = 951; ** *p* ≤ 0.01; *** *p* ≤ 0.001; QD: quantitative demands; FB: feedback.
